# Acute histological reactions in the otolith organs to inner ear drug delivery through a cochlear implant

**DOI:** 10.3389/fneur.2024.1363481

**Published:** 2024-02-26

**Authors:** Raquel Manrique-Huarte, Marta Álvarez de Linera-Alperi, Nicolás Pérez-Fernández, Manuel Manrique

**Affiliations:** ^1^Department of Otorhinolaryngology, Clínica Universidad de Navarra, University of Navarra, Pamplona, Spain; ^2^Department of Otorhinolaryngology, Clínica Universidad de Navarra, University of Navarra, Madrid, Spain

**Keywords:** cochlear implant, vestibular dysfunction, histological vestibular reaction, hydrops, inner ear delivery

## Abstract

**Introduction:**

Cochlear implantation is currently regarded as a safe and minimally invasive procedure. However, cochlear implantation can have an impact on vestibular function, despite the lack of correlation between patient symptomatology and damage in vestibular tests. Thus, the present study aims to analyze the presence of hydrops and histological reactions at the level of the vestibule after cochlear implantation with dexamethasone pump delivery in *Macaca fascicularis* (Mf).

**Materials and methods:**

A detailed histological study was conducted on a total of 11 Mf. All 11 Mf were divided into three groups: 5 Mf were implanted with an electrode array HL-14 connected to a pump delivering FITC-dextran for 24 h (Group A); 4 Mf were implanted with a CI electrode array attached to a pump for FITC-dextran delivery for 7 days (Group B); and 2 Mf were considered the control group, without any kind of cochlear device implantation (Group C). After drug deliver, the selected macaques were euthanized to collect tissue samples for histological analysis. An experienced observer, focusing on the utricle and saccule areas, conducted a blinded inner ear histology analysis.

**Results:**

Surgical procedures were successfully performed in all cases. No signs of cochlear reaction to the device were observed, including neither collapse nor fibrosis. Endolymphatic sinus dilatation was observed in Mf4A and Mf3B, while cochlear hydrops was observed in Mf3A. The mean areas of the utricle and saccule exhibited some statistically significant differences, specifically, in the saccule between groups C and both groups A (*p* = 0.028) and B (*p* = 0.029); however, no significant differences were observed between groups A and B or among comparisons of the utricle.

**Discussion:**

A significant concern relates to the safety of cochlear implantation with regard to vestibular preservation and hearing. New advancements in electrode arrays, such as CI devices coupled with delivery pumps, pose a challenge in maintaining minimally traumatic surgical concept-based procedures without affecting the inner ear homeostasis. The implantation of this device may cause vestibular hydrops in the saccule, indicating that the longer the time of substance release, the greater the grade of hydrops evidenced at the saccular level. Apart from this finding, the risk of histological damage to the vestibule is low.

## 1 Introduction

Cochlear implantation (CI) surgery has undergone significant advancements in the treatment of individuals with severe-to-profound hearing loss over the past 40 years (1). CI is currently regarded as a dependable and safe procedure. The use of atraumatic electrodes and minimally traumatic surgical concepts (MTSC) has enabled the preservation of residual hearing following implantation (2). Even though histological and functional research demonstrate the rates of hearing preservation following implantation, including reimplantation at a 6-month follow-up (3, 4), the impact of cochlear implantation following MTSC on the vestibular function remains unclear (5).

The vestibular system has five sensory receptors located in the inner ear, of which two are located in the vestibule, namely the utricle and the saccule, which detect linear accelerations. The remaining three receptors are situated in the semicircular canals, namely the superior, horizontal, and posterior, which detect angular accelerations. During surgery, the vestibular system can be affected by various complication such as direct trauma caused by electrode insertion, inflammatory reactions to the implantation, foreign body reactions with labyrinthitis, endolymphatic hydrops, and electrical stimulation from the implant (6).

Most of these complications can be resolved surgically, and specific measures are currently implemented to protect residual hearing after surgery. Modifications in the electrode design and improved surgical tips tend to mitigate the loss of inner ear function. However, the potential risk of damaging the vestibular end organ is still present.

In a clinical scenario, the prevalence of vestibular disorders, mainly presented by vertigo or disequilibrium, is wide, ranging from 0.33 to 75% (7), although there is usually no correlation between patient symptomatology and damage in vestibular tests. Functional deterioration of the saccular function and the horizontal semicircular canal have been described (8).

A histopathological study conducted at the vestibule level could significantly contribute to understanding the consequences of placing an electrode array inside the cochlea. This study could help answer questions raised by this procedure, such as what kind of damage might be observed in the vestibule after CI following MTSC. The possibility to study a conventional cochlear implant electrode array associated with drug delivery may shed light on the effect of cochlear implantation in the vestibule. We hypothesize that inner ear hydrops may be induced by drug delivery, depending on the amount or the time of delivery. The present study aims to analyze the presence of hydrops and histological reactions in the vestibule after cochlear implantation with dexamethasone pump delivery in *Macaca fascicularis* (Mf).

## 2 Materials and methods

### 2.1 Overview

**Figure 1:**
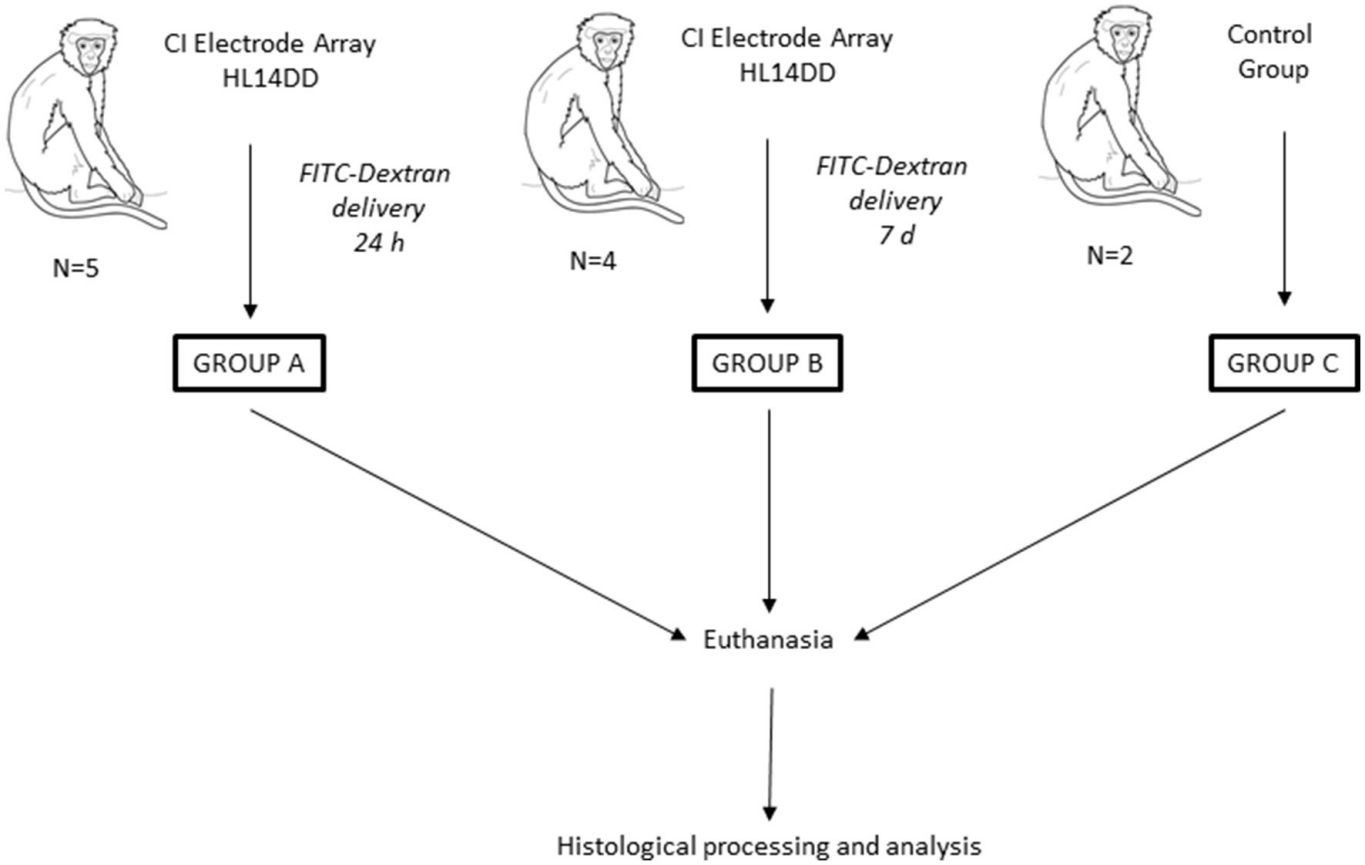
Overview of the study. A total of 11 Mf were included. Three groups were created. Mf were classified into different groups based on the existence of a preclinical device, the Cochlear Ltd. manufactured CI Electrode Array HL14DD, connected to a release pump that was loaded with FITC-dextran and to the programmed release time for group A (24 h) and for group B (7 days). Group C consisted of 2 Mf without any kind of cochlear device. This last group was regarded as the control group. After animal euthanasia, histological processing and analysis were conducted.

In the present study, we conducted a histological analysis after cochlear implantation in *Macaca fascicularis* (Mf). For the study, three groups were established: Group A comprises 5 Mf implanted with a CI electrode attached to a pump delivering FITC-dextran in the cochlea for 24 h, where Mf are labeled as Mf1A, Mf2A, etc. Group B comprises 4 Mf implanted with a cannulated CI electrode and pump, which delivered FITC-dextran in the cochlea over 7 days, where Mf are named Mf1B, Mf2B, etc. Group C comprises 2 Mf that were not implanted with any CI and considered as the control group. The latter 2 Mf were labeled Mf1C and Mf2C. Macaques were sacrificed to collect tissue samples, and the histological analysis was conducted (Figure 1).

### 2.2 Cochlear electrode array

The CI Electrode Array HL14DD, which is a preclinical research array, is manufactured by Cochlear Ltd., Australia. This HL14DD array is employed in groups A and B for intracochlear drug delivery, and it is identical to the previously described HL14 array, with the inclusion of a fluid delivery cannula.

**Figure 2:**
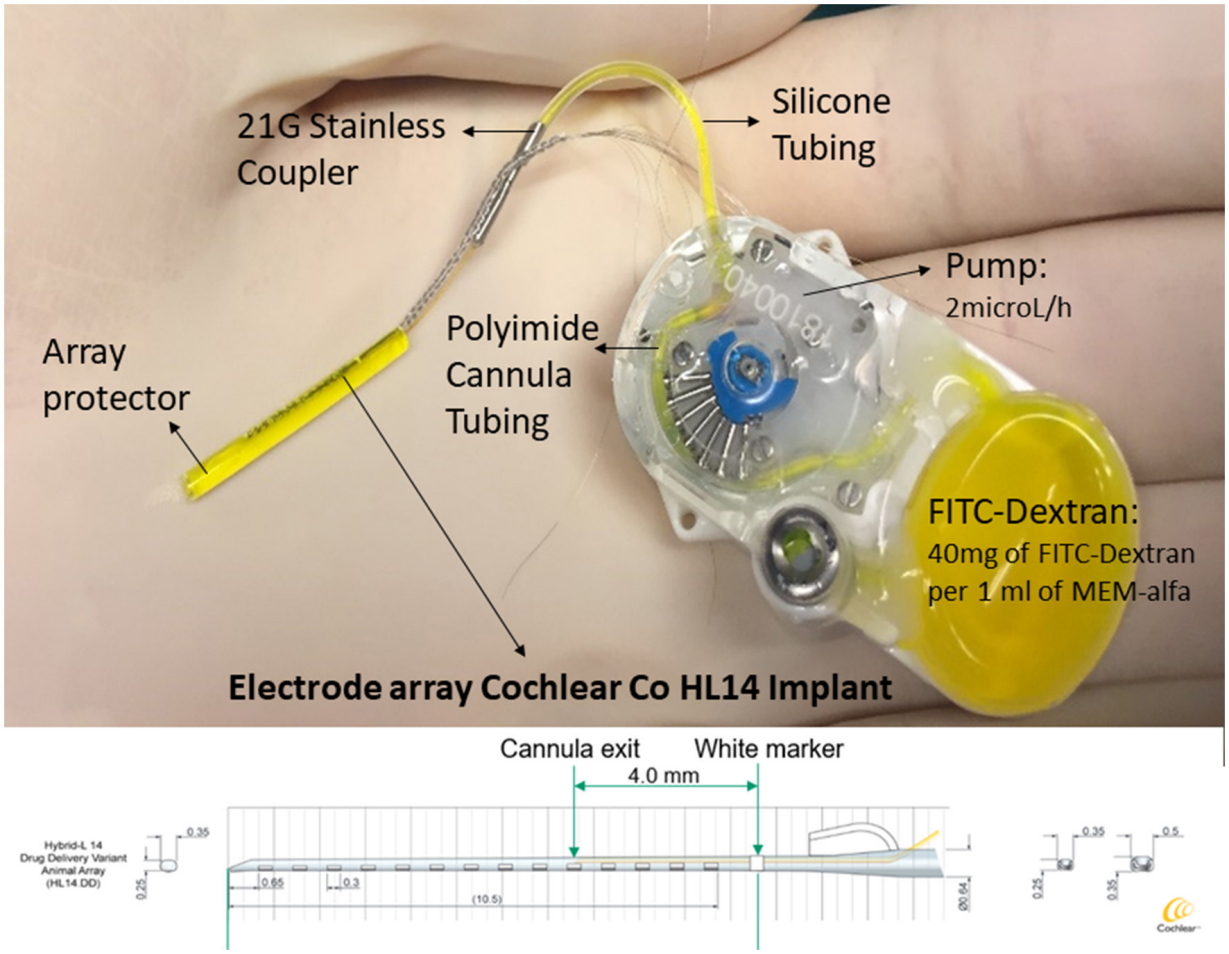
An implantable device and delivery pump system. An HL14 IC electrode array (Cochlear Ltd.), consisting of 14 electrodes with a maximum diameter of 0.5 mm and a total length of 11.5 mm, is the device employed in this investigation. The device comes with a pump that has FITC-dextran loaded into it. This pump is connected to a stainless steel coupler, which is fastened to the electrode array's cannula exit, with a silicone tube. The integrated cannula emerges 4 mm from the basal level white marking. Plotting the depth obtained in the case of a full insertion against the animal's tonotopic distribution, the result is 11 mm, according to Moody et al. (9).

The HL14DD electrode array that includes an integrated cannula that exits 4 mm apical from the white marker is shown in Figure 2. Additionally, this figure illustrates the depth attained in the event of a complete 11.5-mm insertion. Prior to attaching the cannulated electrode array and pump, FITC-dextran was produced under sterile conditions. The following components were combined: the 10 mM concentration of FITC-dextran (fluorescent dextran, FW-4000, Sigma-Aldrich, St. Louis, USA) is 40 μg/ml (MEM α; 51200–038; free of phenol red; Fisher Scientific: www.thermofisher.com). Subsequently, in a sterile environment, the FITC-dextran was injected into the micro-infusion pump (iPRECIO SMP-200^®^, Tokyo, Japan). Using a 1-ml syringe with a 27-Gauge cannula, 1.175 ml of FITC-dextran was roughly injected into the pump. Once the pump was completely filled with FITC-dextran and checked for any trapped air, a stainless steel coupler was connected to the cannula outlet of the electrode array (10). The iPRECIO software was used to program the delivery rate of 2 μl/h before insertion. This delivery rate enables the minimization of the intracochlear longitudinal flow that could be generated by fluid injection through the round window (11). These data are supported by results obtained from a computer program (*Alec Salt's Perilymph World*) developed by Alec Salt's working group that represent drug distribution in the inner ear (https://alecsalt.com/). In addition, this program also allows us to set up the kinetic parameters for the drug molecule based on the molecular properties of the substance. The choice of the substance to be injected is one of the most conditioning factors when studying cochlear pharmacokinetics: “*minor changes in the drug molecule can make huge differences in the physical properties which influence pharmacokinetics, undoubtedly affecting the efficacy of the substance when applied to the ear”* (12). Small lipophilic molecules with few polar groups are those that are able to cross biological membrane barriers more easily. Currently, various substances are being studied for intracochlear drug delivery. An example of this phenomenon is dexamethasone phosphate, which has a higher molecular weight and is less liposoluble because it is more polar than isolated dexamethasone. As a result, dexamethasone phosphate crosses the barriers more slowly and remains in the injected area for a longer time. When dexamethasone phosphate reaches its active form (when it loses phosphate), it becomes less polar and, therefore, crosses biological barriers faster. Although the release of dexamethasone phosphate at the intracochlear level has a *uniform distrib*ution along the tympanic scale, the solutes in the perilymph are distributed to various inner ear tissues, including the spiral ligament, the spiral ganglion, or the canaliculi of the bony walls of the ear, until stability in the distribution between compartments is achieved (13, 14).

FITC-dextran has been demonstrated in previous pharmacokinetic investigations to be well-retained in the perilymph, outperforming other substances examined. Estimations suggest that losses would not be significant enough to change the substance's distribution to the apical regions of the cochlea (15).

### 2.3 Experimental animals

The weight of animals ranged from 1.85 to 4.06 kg. The specimens were housed and treated at the animal facilities of the University of Navarra in accordance with EU Regulation 86/609 and the protocols approved by the University of Navarra's Animal Care and Use Committee (file numbers 083/18 and 001/22).

The selection of this macaque species (Mf) was based on its close evolutionary relationship to humans, which frequently serves as a vital conduit between fundamental studies and practical implications for human health. As previously demonstrated, the surgical method utilized is comparable to that used in human (4, 16). This fact is crucial to achieving the suggested goals and translating the findings to clinical practice.

### 2.4 Anesthesia procedure

According to the procedure outlined for this species, all specimens were anesthetized using an analgesia and closely monitored (17).

An intramuscular dose of a combination of atropine sulfate (0.05 mg/kg), midazolam (0.5 mg/kg), and ketamine (5 mg/kg) was administered to induce mechanical immobility. The animals were administered an IV dose of 10 μg of fentanyl before the initial incision. To maintain general anesthesia during the surgical process, the animals were provided with a mixture of nitric oxide, oxygen (50%), sevoflurane (2–3%), and a fentanyl infusion (2–4 mg/kg/h) or Ultiva (0.5–0.9 μg/kg/min). No systematic administration of corticosteroids occurred during the postoperative and follow-up phases, since this administration may interfere with the natural inflammatory process.

Following the investigation, the animals were put to sleep in accordance with the procedure that the University of Navarra's Committee on Ethics had set forth. For this reason, 200 mg/kg of salt was injected intravenously via pentobarbital, overdosing on barbiturates right before the infusion began. The tree was then cleaned using an intracardiac saline solution and fixed with paraformaldehyde perfusion tissues. A description of the tissue fixation procedure is given as follows: (1) isotonic saline lavage of the vascular tree is the remedy (Ringer's solution); (2) perfusion using a 4% paraformaldehyde cold solution in phosphate buffer (0.1 M, at ambient temperature and pH 7.4) (for a total of three perfusions, the rate of perfusion was 1 L/15 min); and (3) reperfusion using a 10% cryoprotection solution containing glycerin in 0.1 M phosphate buffer and dimethyl sulfoxide (DMSO).

### 2.5 Surgical procedure

The round window membrane approach is the standard method employed in CI surgery to introduce an electrode array. Manrique-Huarte et al. (18) published the details for the implantation of the pump and cannulated system. In brief, following the preparation of FITC-dextran under sterile conditions, the pump is filled into the micro-infusion pump (iPRECIO SMP-200^®^, Tokyo, Japan) using a 1-ml syringe with a 27-Gauge cannula. Once the pump was filled and checked for trapped air, the stainless steel coupler (SS coupler) was connected to the cannula outlet of the electrode array. The device was placed following the same steps as a standard cochlear implant surgery. The delivery rate was programmed using iPRECIO software prior to insertion.

For groups A and B, follow-up surgeries were conducted 24 h and 7 days following implantation, respectively. These surgeries involve cleaning out the previous incision and removing any fibrous tissues. We used a drill with a 1.5-mm diameter and a speed lower than 4,000 rpm to drill the bone layer of the apical region of cochlea. The drilling process preserved the last layer of the endosteal layer. Following the drilling process, the interior wall of the tympanic cavity was meticulously dried. Once this goal was accomplished, using the procedure outlined by Salt et al. (19), cyanoacrylate was placed. A silicon cup was made by *Kwik-Cast World Precision Instruments in Sarasota, Florida*. In the final stage of the apical cochleostomy, the cochlea was opened and the residual boneshell was removed using a pick angle (Storz 1/3mm 30^*^ *House stapes pick N1705 80, Bausch and Lamb Inc*.) without interrupting the action of pump. At this stage, the perilymph outflow could be observed. Subsequently, a total of 10 samples were collected using capillary tubes (*Blaubrand*^®^ *Ref 708707. Wertheim, Germany*), with at least 1 ml extracted from each sample. To preserve FITC-dextran's fluorescent qualities, samples were placed into dark Eppendorf tubes (*Sartstedt AG& Co. Numbrecht, Germany*) before analysis. While the cable, cannula, and electrode array remained in place for additional histological analysis, the pump was removed.

### 2.6 Temporal bone extraction and histological processing

Using the traditional protocol employed in temporal bone laboratories, the petrosal ridges were extracted for histological processing (20).

Following their extraction, the specimens were dried out and set in epoxy resin (21). After taking pictures of the resin block and a temporal bone, the electrode array was left in place. The sample was then sectioned, with serial slices spaced every 100 μm, dyed with toluidine blue, and photographed using a stereoscopic microscope (Leica^®^S8AP) at magnifications of 1.25-, 1.6-, 2-, 4-, and 6.3-fold for the posterior analysis of histological findings.

### 2.7 Histological analysis

An experienced observer, focusing on the utricle and saccule regions, conducted a thorough blinded inner ear histological analysis to evaluate the impact of cochlear implant surgery.

**Figure 3:**
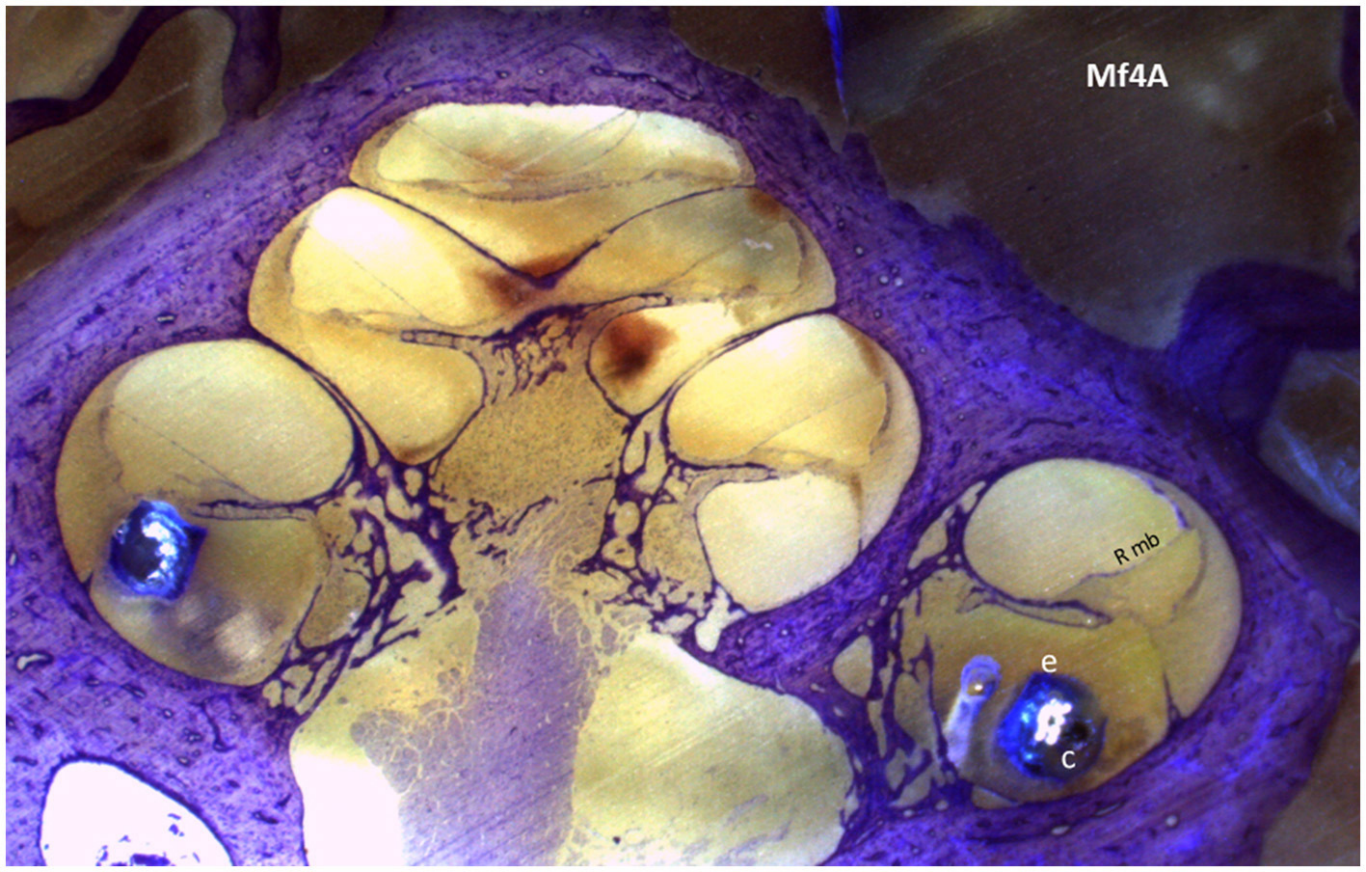
A section of the cochlear mid-modiolar view of the cochlea. The electrode array (e) is in the scala tympani in the basal turn; within the array, the cannula (c) is located in the first 4 mm of the array. Rmb, Reissner membrane.

**Figure 4:**
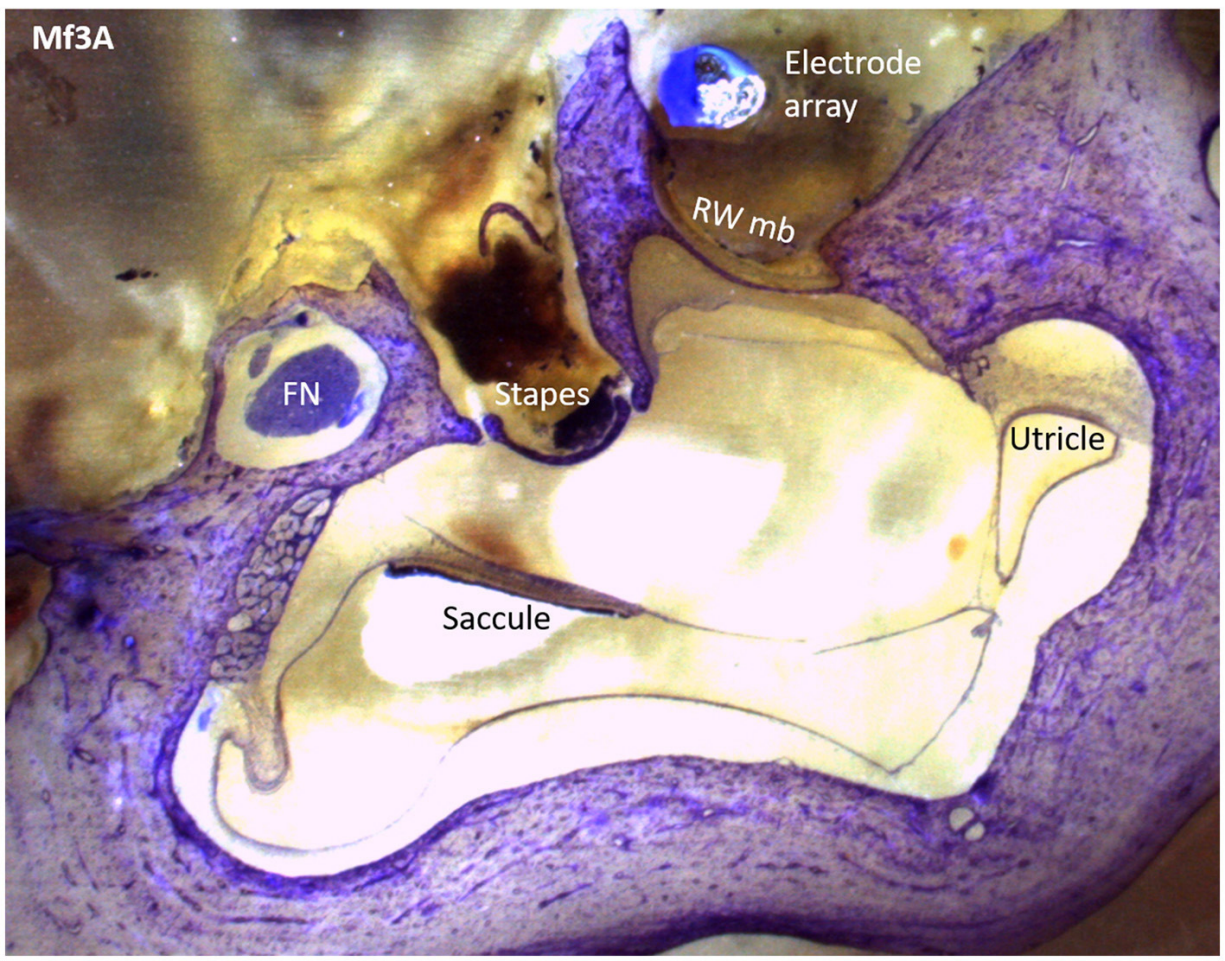
A Mf3A histological section that shows the saccule, utricle, and surrounding structures with epoxy and toluidine blue staining.

To find the presence of tissue reactions, including fibrosis, saccule or utricle collapse, and ossification, we first examined the anatomy of the saccule and utricle. Following appropriate preparation, the specimen was carefully placed in the polisher to produce serial sections that, upon passing through the cochlea, presented a mid-modiolar view (Figure 3). The vestibule's saccule and utricle regions were located, as shown in Figure 4, and their areas were measured to determine the presence of hydrops.

Using ImageJ FIJI software (22), the saccule and utricle areas were measured while considering the pixels of their membranes in the images. These measurements were taken by selecting from the histology pictures of macaques' anatomy that showed the saccule or utricle. With these measurements, the mean values were calculated. We evaluated the intracochlear structures by assessing the presence or absence of cochlear hydrops, measuring the displacement of the Reissner membrane in the basal membrane and therefore the presence of cochlear hydrops.

The mean of the results (standard deviation, SD) is displayed. The means of the two groups were compared using the Student *t*-test (two-tailed), with a *p*-value of <0.05 being regarded as statistically significant (SPSS 20.0, IBM).

## 3 Results

The surgical procedures were successfully performed on all Mf. No complications during surgical and anesthetic procedures were observed. A histological processing and analysis were conducted, and the findings are summarized in Table 1.

**Table 1:** Histological findings for each *Macaca fascicularis*.

**Mf**	**Cochlear hydrops**	**Vestibule histological findings**	**Cochlear histological findings**
Mf1A	No	No	No
Mf2A	No	No	No
Mf3A	Yes	No	No
Mf4A	No	Endolymphatic sinus dilatation	No
Mf5A	No	No	No
Mf1B	No	No	No
Mf2B	No	No	No
Mf3B	No	Endolymphatic sinus dilatation	No
Mf4B	No	No	No
Mf1C	No	No	No
Mf2C	No	No	No

### 3.1 Vestibular assessment

**Figure 5:**
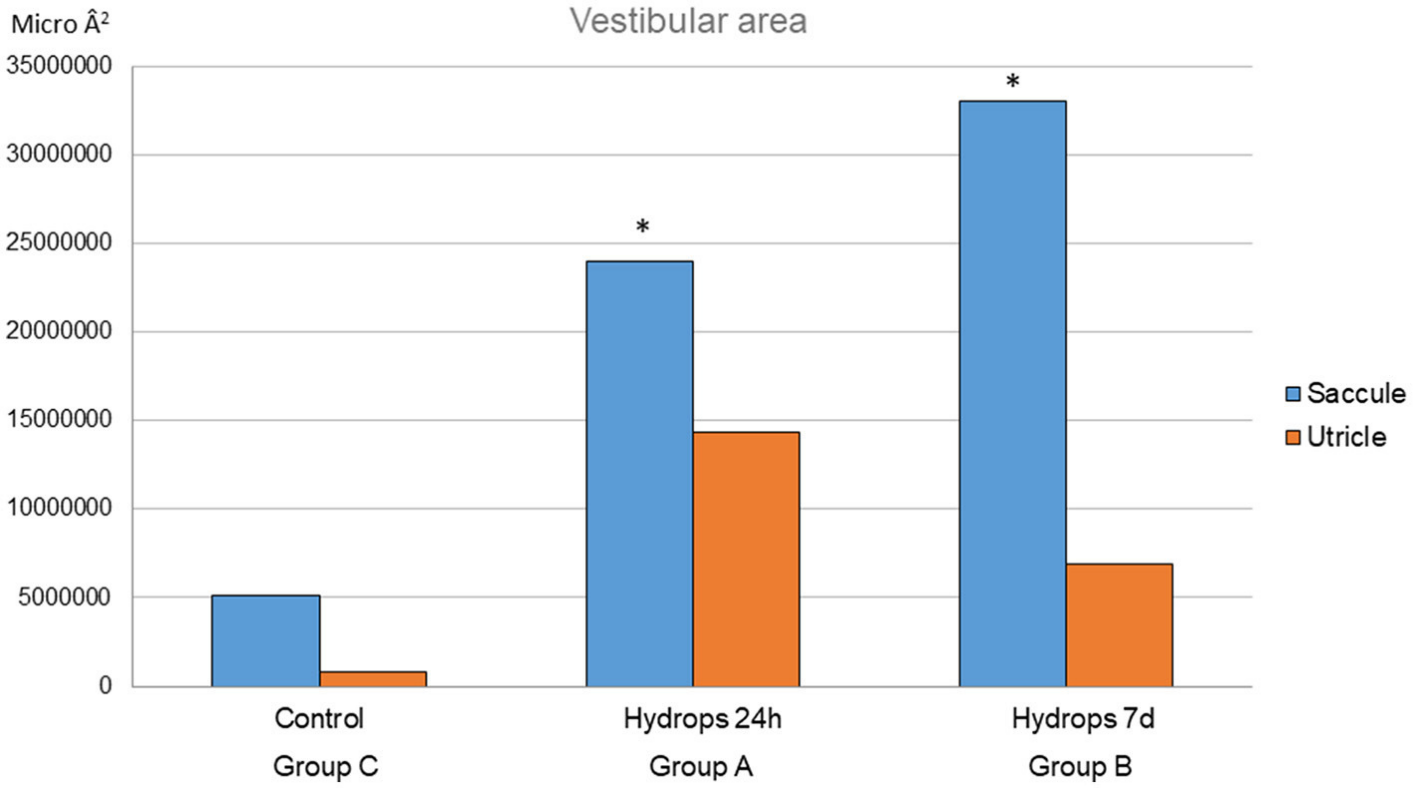
This graph represents the utricle and saccule areas for each group. For the saccule mean area, there are statistical differences between the control group and groups A and B (*p* = 0.028 and *p* = 0.029, respectively). ^*^Statistical significant differences found.

Regarding vestibular end organ reaction to the cochlear implant, signs of endolymphatic sinus dilatation were observed in Mf4A and Mf3B. For volume quantification of hydrops, in group A, the mean saccule area is 24025768.15 Â^2^ (SD 7809036.11) and the mean utricle area is 14,376,730.77 Â^2^ (SD 4,322,316.885). In group B, the mean saccule area is 33,077,442.27 Â^2^ (SD 14,775,493.54) and the mean utricle area is 6,871,518.59 Â^2^ (3,919,129.22). In group C, the mean saccule area is 5,099,298.59 Â^2^ (SD 5,402,388.14) and the mean utricle area is 8,474,000 Â^2^ (Figure 5). Statistical differences were observed in the saccule area between groups A and C (*p* = 0.028) and also between groups B and C (*p* = 0.029). Between groups A and B, no statistically significant differences were observed in the saccule area (*p* = 0.273). There were no statistically significant differences between any of the groups in terms of the mean utricle area (see Figure 5).

### 3.2 Cochlear assessment

**Figure 6:**
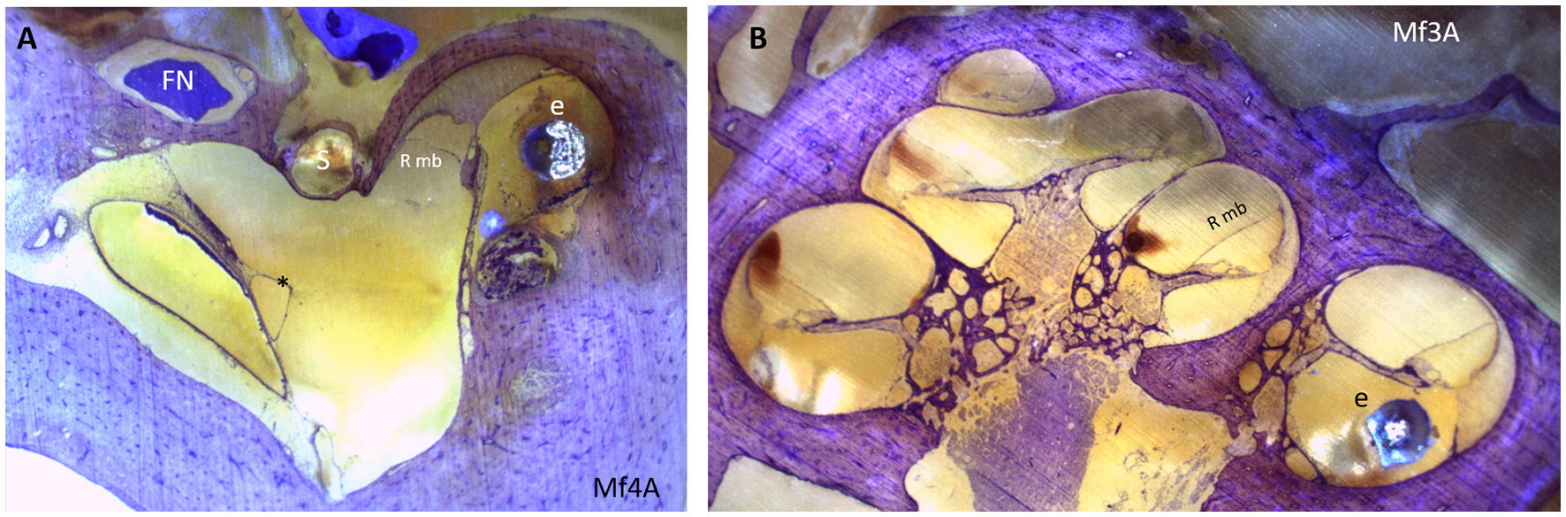
**(A)** Endolymphatic sinus dilatation (*). **(B)** A mid-modiolar view shows displacement of Reissner membrane and therefore the presence of cochlear hydrops. An electrode array is placed in the scala tympani. e, electrode array; FN, facial nerve; Rmb, Reissner membrane; S, stapes.

For group A, there are signs of Reissner membrane distension in Mf3A (Figure 6) and cochlear hydrops in one out of the five Mf. No signs of hydrops are depicted among groups B and C.

Considering the apical cochleostomy performed in Mf from groups A and B, signs of the procedure are observed, including the partial occupation of apical turns with bone dust [see Manrique-Huarte et al. (18) for more details].

## 4 Discussion

Currently, cochlear implant is the primary line of treatment for severe-to-profound sensorineural hearing loss. Although the results of the hearing test are quite good, the extent of the structural and functional harm caused by the insertion of such a device remains uncertain. The current study examines the safety of cochlear implantation with regard to the preservation of the vestibular and hearing functions following MTSC and the use of various electrode arrays (23). Recent advancements in cochlear implants, including those enabling the local intracochlear delivery of protective molecules or the type of electrode array used, may still exert pressure changes in the cochlear fluids, potentially resulting in damage to the surrounding structures (24–27). Some authors have identified the sacculus as mostly affected, with histopathological reports describing distortion of the saccular membrane, partial or complete collapse, or in some instances, hydropic distention (8, 28). A reasonable assumption is that the saccule will be more vulnerable than the utricle or semicircular canals due to its near proximity to the direction and course of the electrodes positioned there. The susceptibility to stress is also influenced by the physical properties of the sacculus walls. Research has indicated that the sacculus and Reissner's membrane exhibit the highest susceptibility to stress (29). A shared pathway has been observed between the ductus reuniens and the cochlear duct (CD), which can become blocked by the fibrous tissue or bone fragments. This phenomenon can also result in the collapse of the dependent saccule (8, 30). Previous research conducted by our group suggests that cochlear insertion of an electrode array following MTSC principles after 6 months of follow-up provokes histologically minimal changes in the vestibule and signs of endolymphatic sinus dilatation and saccule collapse (31). However, no major signs of damage, such as ossification, fibrosis, or neuroma, are observed.

The results of this study show the histological findings obtained at the vestibule, with an analysis conducted across three different scenarios. In group A, recruited Mf received a supply of FITC-dextran in the cochlea through an implanted cannulated CI electrode and an active pump programmed to deliver 2 μl/h during a period of 24 h using iPRECIO software. In group B, selected Mf received a supply of FITC-dextran in the cochlea through the same electrode array employed in group A and an active pump programmed to deliver the supply of 2 μl/h over a 7-day period using software. In group C, selected Mf were identified without any kind of cochlear device implantation. According to previous studies, no major signs of damage in the vestibule were observed. The quantification of hydrops in the saccule shows statistically significant differences between groups A and B compared to group C. These results may support the hypothesis that either the electrode array insertion pathway, a blockage of the pathway between the ductus reuniens and the cochlear duct, or a foreign body reaction to the device may induce acute changes in the saccule. According to our research, the administration of FITC-dextran may also influence inner ear homeostasis, which could contribute to the formation of hydrops in the saccule. The fact that the sampling and euthanization differ between groups A and B, varying in time frame from 24 h to 7 days, might shed light on the acute time course of hydrops. In both groups, the dimensions of the saccule are higher than that in the control group. Therefore, the injection of FITC-dextran may provoke, in acute stages, a hydropic reaction in the saccule, despite the collection of a 10-μL sample of perilymph after 24 h and 7 days of intracochlear delivery.

The inner ear, embedded in the temporal bone, is filled, under physiological conditions, with fluid (32) in a non-distensible compartment. This fact is largely conditioned by the bony layer that covers the inner ear known as the otic capsule. Moreover, to maintain the adequate functioning of the sense organs, an adequate homeostasis between the labyrinthine fluids endolymph and perilymph is required. This homeostasis is physiologically carried out through structures such as the blood–labyrinthine barrier or the communication existing between the cerebrospinal fluid and the perilymph or endolymph. These fluids are primarily connected by the cochlear aqueduct or the endolymphatic sac, respectively (13, 33). An example highlighting the importance of maintaining adequate homeostasis would be Meniere's disease. The symptoms include auditory fluctuations, with gradual sensorineural hearing loss and the impression of otic fullness, tinnitus, and repeated episodes of vertigo lasting from minutes to hours. The histopathological hallmark of the disease, whose etiology and pathophysiology are still unknown, is endolymphatic hydrops, which are conditioned by changes in endolymph–perilymph regulation and influenced by internal and external factors, such as hormonal or barometric pressure changes or stress (34, 35).

Following these premises, this research enables the study of two main aspects in relation to iatrogenic vestibular hydrops as follows: (1) the influence of the placement of a cochlear implant in the scala tympani and (2) the influence of substance delivery directly at the intracochlear level associated with a cochlear implant.

Therefore, on the one hand, because implantation of a CI device in the scala tympani entails occupying some space within that structure, it always results in a shift in inner ear pressure, which can cause hydrops to some extent. Recent research has demonstrated an incidence of 42–59% of cochlear endolymphatic hydrops in relation to cochlear implantation (36). However, our results refute these percentages in the very initial stages of follow-up. Thus, while analyzing the results obtained in the 9 Mf implanted (the ones belonging to groups A and B), this incidence drops by as much as 11%. It is important to consider that the guinea pig experimental models of endolymphatic hydrops damaged by the endolymphatic sac show initial functional or structural damage 1–4 weeks after upgrowing since then (37, 38). We have to consider another limiting factor for comparison as one of our manipulations: the substance release performed 24 h or 7 days after implantation could potentially be a source of added damage. Finally, for the control group, group C, no evidence of cochlear hydrops was detected. Further research is needed to verify these findings with a longer duration of follow-up and the possible influence of inner ear drug delivery and sampling in cochlear hydrops.

On the other hand, the importance of inner ear homeostasis has similarly been demonstrated when injecting any type of drug into the inner ear, in terms of both efficacy and potential damage to this sense organ (39). Taking these concepts into account, we hypothesize that introducing a certain volume of substance in the cochlea produces histological hydrops in both the cochlea and vestibule. Thus, the comparison made in this study between those specimens in which the cochlea was kept intact (Group C) without any type of implantation, and those Mf that were implanted with an intracochlear device associated with a delivery pump (Groups A and B) showed statistically significant differences in the measurements obtained in the sacculus after a delivery period of at least 24 h. The degree of saccular hydrops increases as the release time extends up to 7 days. These hydropic differences could not be demonstrated when comparing different FITC-dextran release times (Groups A and B). Nevertheless, the decrease in utricular hydrops shown in Group B (Figure 5) as the time of substance release increases (7 days) may be connected to the endolymphatic sac's regulation of pressures, even though there were no statistically significant differences found in relation to the utricular mean area among the three groups. This fact has previously been described and related to the closer proximity of this structure to the utricle. According to estimates, the concentration profile starts to decrease after at least 24 h to 7 days of release time in Mf, which coincides with the onset of the “washout” period of drugs (40). The appropriate choice of the injectable material (FITC-dextran is known to be effectively held in the perilymph), dosage, and release flow across the cochlea are key factors in maintaining homeostasis and, subsequently, the presence or absence of iatrogenic hydrops. In this case, the delivery rate was programmed as 2 μl/h. This flow rate had been previously calculated to reach the most adequate dose to achieve good distribution within the cochlea. However, it is also very important to know in depth the elimination pathways of the substance, not only to prevent possible dysregulation of the labyrinth fluids with the subsequent iatrogenic endolymphatic hydrops but also to assess a possible toxicity effect at the cochlear and vestibular levels (40). Moreover, it is widely recognized that there is not always a direct correlation between endolymphatic hydrops and the symptomatology experienced by the patient (41, 42). To this extent, the histological findings evidenced in this study show only some kinds of hydrops, not necessarily related to functional outcomes, and additional research ought to be done to objectively evaluate the proper performance of the vestibular end organs following inner ear drug delivery. Furthermore, the results we obtained are based on a brief follow-up period; a longer follow-up period may yield various findings. These variations could result from altered labyrinth fluid regulation, and local inflammatory responses that trigger additional tissue growth (43) altered inner ear homeostasis or both. To clarify the physiopathological mechanisms behind these changes in both acute and chronic scenarios, further research is warranted.

Finally, it is critical to consider the similarities in anatomy and physiology between the species under study, Mf, and human beings. Some of these findings can be extrapolated from one study to another due to their close evolutionary relationship (32, 44). However, it is always crucial to remember that there can still be some specific differences between the two specimens, even when they are phylogenetically close.

## 5 Conclusion

The findings reported in this study support the following assertions:

- No major signs of vestibular damage are observed after cochlear implant with substance delivery.
- Injecting FITC-dextran via a cochlear implant for at least a 24-h period enables the histological examination of endolymphatic hydrops in the saccule.
- The longer the time of substance release, the greater the hydrops evidenced at the saccular level.

## Data availability statement

The original contributions presented in the study are included in the article/supplementary material, further inquiries can be directed to the corresponding author.

## Ethics statement

The animal study was approved by Comite de etica del instituto de salud pública y laboral de Navarra. The study was conducted in accordance with the local legislation and institutional requirements.

## Author contributions

RM-H: Conceptualization, Investigation, Methodology, Writing – original draft. MA: Formal analysis, Investigation, Methodology, Writing – original draft. NP-F: Conceptualization, Supervision, Validation, Writing – review & editing. MM: Project administration, Resources, Supervision, Writing – review & editing.
